# A comparative analysis of hematopoietic stem cell transplantation in pediatric and adult patients: a systematic review and meta-analysis

**DOI:** 10.3389/frtra.2025.1551820

**Published:** 2025-06-05

**Authors:** Shailendra Shanker Maurya, Nitin Sagar, Sumit Chaturvedi, Maneesha Pandey, Sapana Kushwaha, Rajesh Kashyap

**Affiliations:** ^1^ Stem Cell Research Center, Department of Hematology, Sanjay Gandhi Postgraduate Institute of Medical Sciences, Lucknow, India; ^2^ Department of Biophysics, University of Delhi South Campus, New Delhi, India; ^3^ School of Medicine, Washington University Saint Louis, St. Louis, MO, United States; ^4^ Department of Pharmacology and Toxicology, National Institute of Pharmaceutical Education and Research (NIPER) - Raebareli, Lucknow, India; ^5^ Department of Hematology, Sanjay Gandhi Postgraduate Institute of Medical Sciences, Lucknow, India

**Keywords:** acute myeloid leukemia, confidence interval, odd ratio, relapse rate, treatment related mortality, overall survival, disease free survival

## Abstract

**Introduction:**

Hematopoietic stem cell transplantation (HSCT) is a significant treatment option for acute myeloid leukemia (AML). However, some important questions remain related to its efficacy and safety, specifically when administered to various age cohorts among pediatric and adult patients.

**Aim:**

This study aimed to investigate the efficacy of HSCT in treating pediatric patients compared to adult patients diagnosed with AML.

**Methods:**

A systematic search was conducted in PubMed, Scopus, Google Scholar, and Medline for studies published in the English language from inception to 2023. The findings were reported using the PRISMA checklist. Statistical analysis was conducted using Cochrane's software (Rev Man) version 5.4, which used random and fixed effect models when necessary.

**Results:**

In total, 14 studies met the criteria for meta-analysis. The results indicated a slightly positive trend in overall survival in the pediatric and combined pediatric–adult groups compared to adults alone, although the differences were not statistically significant. For relapse rate, no significant differences were observed in the adult and pediatric groups individually, while the combined pediatric–adult group showed a substantial benefit from HSCT (OR: 2.3, *P*-value: −0.05). A similar trend was observed in disease-free survival, where the combined group showed a modest, though not statistically significant, improvement with HSCT. Furthermore, regarding treatment-related mortality, a statistically protective effect of HSCT was observed in the adult group (OR: 0.26, *P* = 0.0005), while the pediatric and combined groups did not show significant effects. For graft-vs.-host disease, a significant association with HSCT was found in the pediatric group (OR: 2.58, *P* = 0.03), while the adult and combined groups showed no significant effects.

**Conclusion:**

Our analysis showed mixed results, showing a slightly better effect of HSCT in treating pediatric patients diagnosed with AML compared to adult patients.

## Introduction

Acute myeloid leukemia (AML) encompasses a diverse range of malignancies distinguished by the excessive proliferation of myeloid precursor cells in the bone marrow (1). According to Deng et al. (2), abnormal cell growth disrupts the normal process of blood cell formation, resulting in defective hematopoiesis and subsequent systemic manifestations. AML, as stated by Montoro et al. (3), constitutes a large number of patients diagnosed with leukemia among various age groups, exhibiting a wide spectrum of diversity in its clinical manifestation, responsiveness to treatment, and long-term outlook. The effective management of AML requires implementing a multidisciplinary strategy encompassing many treatment modalities, including chemotherapy, targeted therapies, and hematopoietic stem cell transplantation (HSCT), and these modalities play crucial roles in controlling the disease and offering the possibility of a cure.

Historically, the management of pediatric AML has been primarily based on data generated from adult AML studies due to the higher number of adult cases available for clinical trials, and it was assumed that similar biology exists across age groups. However, genomic profiling of AML in both age groups (pediatric and adult) has suggested that AML is a disease with distinct age-dependent alterations (5). Recent studies of pediatric AML genomes indicated substantial differences between the genomic landscapes of adult and pediatric AML (6). Many of the novel and clinically critical genomic alterations identified in adult AML by whole genome sequencing are not observed in childhood AML (7), suggesting a distinct age-associated biology and highlighting the need for genomic profiling of the disease in children.

However, HSCT has emerged as an important treatment option for AML, especially in instances of patients with relapsed/refractory disease or recurrence, or in high-risk disease, where standard chemotherapy fails to achieve a durable remission. Despite its associated risks, HSCT remains a cornerstone of treatment in these patient populations. The intervention presents the possibility of attaining prolonged remission and, in some instances, a prospective remedy (8). The therapeutic approach entails the administration of hematopoietic stem cells, which are commonly obtained from the bone marrow, peripheral blood, or cord blood of a compatible donor, and the primary objective of this intervention is to restore the patient's hematopoietic system and eliminate leukemic cells (9). According to Chao et al. (10), HSCT is acknowledged as a significant therapy alternative for AML. However, some important questions remain related to its efficacy and safety, specifically when administered to diverse age cohorts among pediatric and adult patients.

Recent studies investigated several molecular and cellular factors affecting HSCT outcomes in patients with AML. It was observed that pre-transplant minimal residual disease (MRD) status was a strong indicator of relapse and survival (11). Furthermore, specific genetic aberrations, such as NPM1, FLT3-ITD, and TP53, are also responsible for differential responses to HSCT (12). Likewise, graft source, donor type, and immune reconstitution kinetics also play a role in relapse risk and the graft-vs.-leukemia (GVL) effect (13, 14). Considering these factors and cellular mechanisms has become crucial during the treatment regimens for risk stratification and personalized transplant approaches.

Furthermore, a deeper understanding of the intrinsic/extrinsic regulatory mechanisms of HSC self-renewal and lineage commitment is essential for improving HSCT outcomes. Recent studies have explored how signaling is crucial, as pathways such as Notch, Wnt, and PI3K/AKT/mTOR orchestrate HSC fate, while epigenetic regulators play a role in fine-tuning transcriptional programs critical for maintaining stemness and balanced differentiation (15). Targeting these pathways may promote better hematopoietic reconstitution post-transplant.

This study explores the different age-associated patterns in the clinical parameters of AML, as the responsiveness of treatment regimes and the biology of the illness have triggered ongoing debate and inquiries. Hence, it is essential to diligently assess the available literature to determine whether there are notable disparities in the results of HSCT for AML when comparing the pediatric and adult populations (16).

This systematic review and meta-analysis seeks to evaluate and synthesize findings from studies conducted across diverse regions and clinical environments. The primary focus is to assess the key outcomes of HSCT, including overall survival (OS), relapse rate (RR), and treatment-related mortality (TRM). Furthermore, the analysis will extend to secondary outcomes such as the incidence of graft-vs.-host disease (GVHD) and disease-free survival (DFS). Pochon et al. (17) suggested it is imperative to possess a comprehensive comprehension of the intricate distinctions between pediatric and adult populations within the realm of HSCT for AML to enhance treatment options and improve the quality of health care provided to patients. By highlighting key clinical differences in treatment outcomes, this study aims to offer meaningful insights that can support clinical decision-making and enhance strategies for risk stratification.

## Methods

### Literature search

**Figure 1:**
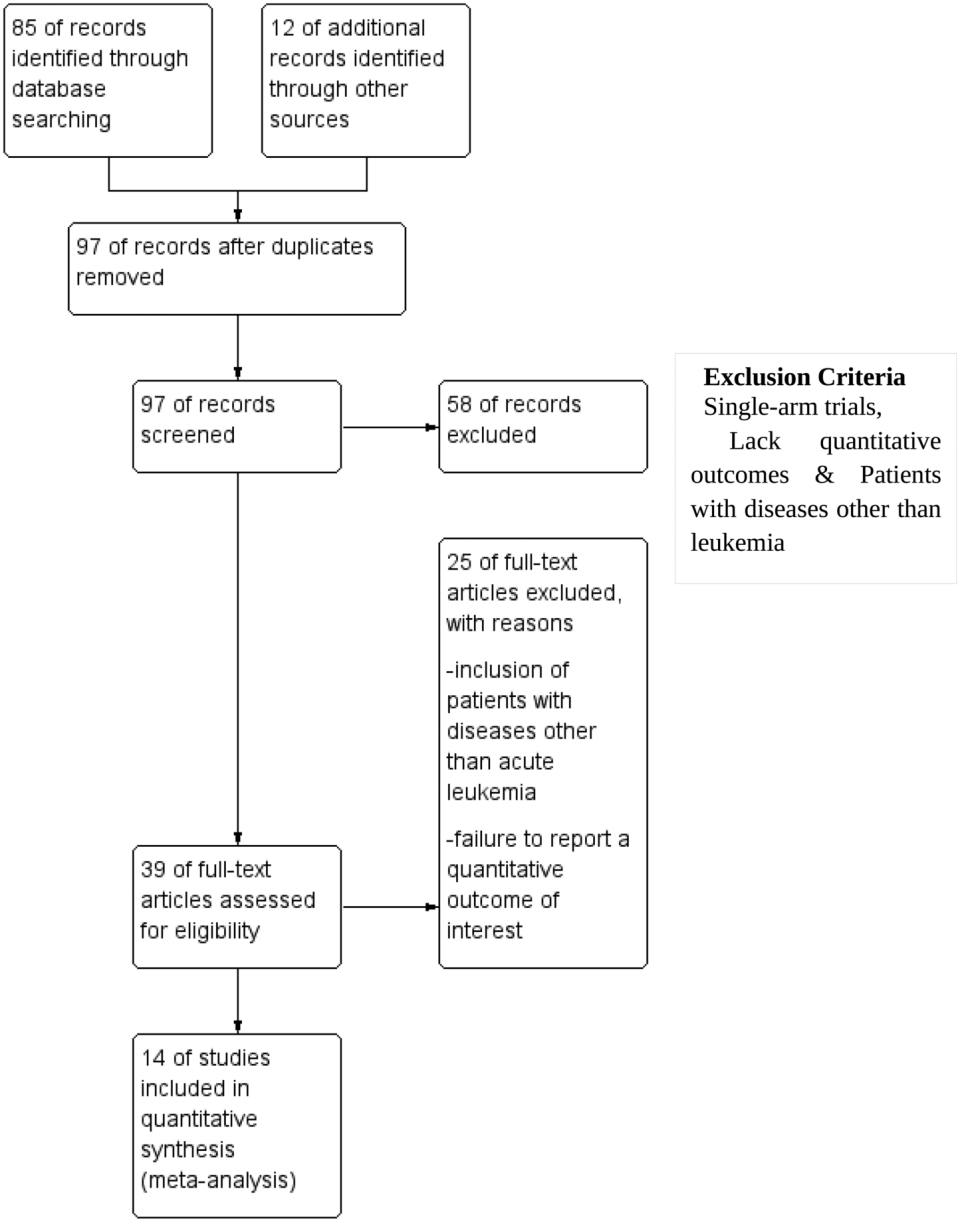
Preferred Reporting Items for Systematic reviews and Meta-Analyses (PRISMA) flowchart for study inclusion.

The methodologies used in this reporting are per the Preferred Reporting for Systematic Reviews and Meta-analysis (PRISMA) standards for performing systematic literature reviews. This systematic review was conducted with the PRISMA 2020 guidelines. A formal protocol was not pre-registered for this review. A comprehensive literature search was performed using Medline, Cochrane, PubMed, Scopus, and Google Scholar databases from inception till December 31, 2023, using specific keywords such as overall survival, disease-free survival, mortality, relapse, acute myeloid leukemia, HSCT, hematopoietic stem cell transplantation, children, adult, and pediatric. Efforts were made to enhance coverage via Google Scholar via a comprehensive search strategy, including gray literature. To ensure consistency in data interpretation, only studies published in English were considered for inclusion. This search aimed to locate relevant publications that have been published up until now. Following the removal of redundant references, our research methodology utilized a two-tiered approach. All discovered publications' titles and abstracts were initially screened based on the predetermined inclusion and exclusion criteria. The articles that satisfied the predefined criteria for inclusion but did not meet the criteria for exclusion were chosen for the subsequent screening stage. During this phase, the complete text of each item was thoroughly evaluated based on the predetermined criteria for inclusion and exclusion. Figure 1 shows the systematic design of the literature search and the study's inclusion.

### Study selection

The inclusion criteria for the study were as follows: (1) The study had to be a randomized controlled trial, a 2-arm study, or a prospective or retrospective study; (2) the study had to involve two different age groups of patients, i.e., adult vs. pediatric patients with acute leukemia who had undergone a transplant with HSCT, with different parameters compared; (3) the analysis must have focused on assessing quantitative outcomes by calculating odds ratios for key parameters, including RR, GVHD, TRM, OS, and DFS. Studies were excluded from the evaluation if they met either of the following limitations: they were single-arm trials or did not report the quantitative outcome measures.

### Outcomes

The key outcomes of this study included overall survival, relapse rate, and treatment-related mortality.

### Data extraction

The data obtained from the included studies encompassed several variables, including the first author's name, year of publication, study design, participant numbers in each group, mean age/age group, follow-up duration, relapse rate, and overall survival.

### Quality assessment

**Figure 2:**
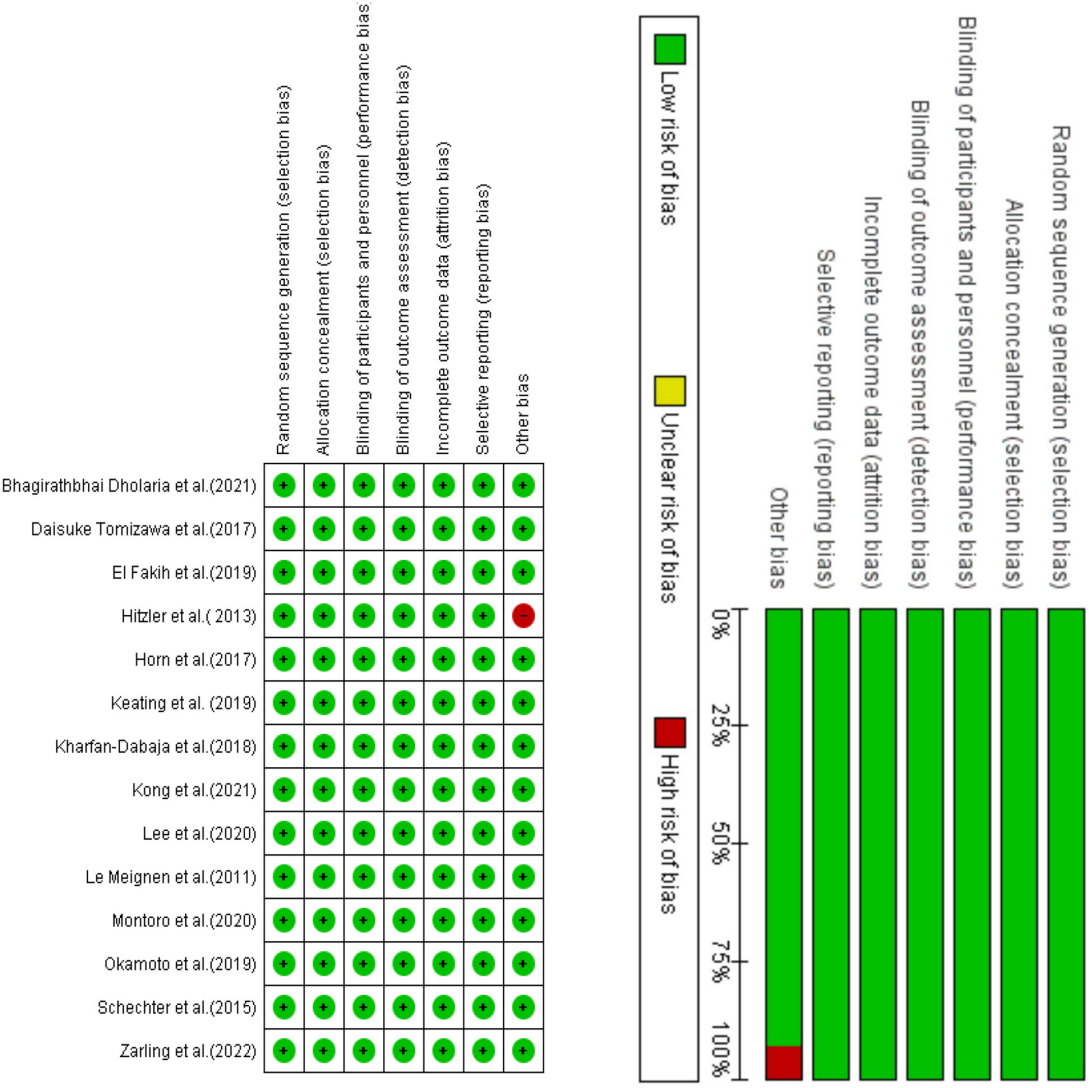
The summary of the reported assessment of the included studies.

The Cochrane Risk of Bias method was employed to evaluate potential bias in the studies included in our analysis, utilizing Review Manager version 5.4 software (18). The technique assesses the possible bias within six areas of a research study, including study attrition, participation, and confounding; outcome measurement; prognostic factor assessment; and statistical analysis and reporting. The level of agreement with a statement that characterizes the overall quality indicates the quality for each source of bias. The presence of green circles and a symbol denoting agreement in Figure 2 signifies that the study was deemed to possess a high level of quality and was devoid of bias. Conversely, red circles and a symbol indicating disagreement in Figure 2 suggest that the study may contain bias.

### Statistical analysis

Each result was compared between the HSCT group and the comparison group. Odds ratios (ORs) along with 95% confidence intervals (CIs) were calculated to evaluate the distribution of all the outcomes. To assess heterogeneity among studies, Tau2 statistics were employed, and the inconsistency index (*I*^2^) was determined for each analysis. An *I*^2^ value exceeding 50% indicated significant heterogeneity, in which case a random-effects model was utilized for meta-analytical estimation. Alternatively, a fixed-effect model was utilized. We computed pooled effects and deemed a two-sided *P*-value of less than 0.05 statistically significant. Additionally, subgroup analyses were conducted to ascertain the combined impact according to patient type (adult or pediatric). Publication bias analysis was conducted using a funnel plot (Figure 4), and all the studies were assessed utilizing the Review Manager software.

## Results

A comprehensive search was conducted across many databases to find studies that compared HSCT to other interventions in patients diagnosed with AML. The scope of the search encompassed manuscripts published up to the present moment. The search strategy, outlined in Figure 1, initially yielded 85 articles. An additional 12 studies were identified through supplementary sources. After a detailed screening of titles and abstracts, 58 articles were excluded because they did not meet the eligibility criteria. After a comprehensive examination of the complete texts of the remaining 39 articles, it was determined that 25 were ineligible for inclusion in the study. The reasons for exclusion were as follows: inclusion of patients with diseases other than acute leukemia and failure to report a quantitative outcome of interest. All the remaining 14 studies included in the systematic review and meta-analysis met the qualifying criteria. The characteristics of all 14 included studies are summarized in Table 1.

**Table 1:** Characteristics summary of studies.

Study	Design	Country	Age group	Follow-up	Outcome
Bhagirathbhai Dholaria et al. (2021) (19)	Retrospective	USA	≥18 years	nr	OS, GVHD, RR, DFS
Daisuke Tomizawa et al. (2017) (20)	Retrospective	Japan	0–29 years	5 years	OS, TRM
El Fakih et al. (2019) (21)	Retrospective	Saudi Arabia	14–22 years	46 months	RR, OS, DFS, TRM, GVHD
Hitzler et al. (2013) (22)	Retrospective	USA	3 years	47 months	OS, RR, TRM
Horn et al. (2017) (23)	Retrospective	USA	12 years	63 months	OS
Kharfan-Dabaja et al. (2018) (24)	Retrospective	nr	≥18 years	5 years	OS
Lee et al. (2020) (25)	Retrospective	USA	15–39 years	3 years	OS, DFS
Le Meignen et al. (2011) (26)	Prospective	France	23 years	14.7 years	GVHD, OS
Montoro et al. (2020) (3)	Retrospective	USA	≥18 years	5 years	GVHD, RR, OS, DFS
Okamoto et al. (2019) (4)	Retrospective	Japan	13 years	1,052 days	OS, GVHD, TRM
Keating et al. (2019) (27)	Retrospective	USA	10 years	4.74 years	GVHD, RR
Schechter et al. (2015) (16)	Retrospective	Canada, USA, Israel	10.2 years	2 years	OS, DFS, RR
Zarling et al. (2022) (28)	Retrospective	USA	≥18 years	3 years	RR, DFS, OS, TRM
Kong et al. (2021) (29)	Prospective	South Korea	0–72 years	5.7 years	TRM

OS, overall survival; TRM, treatment-related mortality; GVHD, graft-vs.-host-disease; DFS, disease-free survival; RR, relapse rate; nr, not reported.

### Effects of the interventions

**Figure 3:**
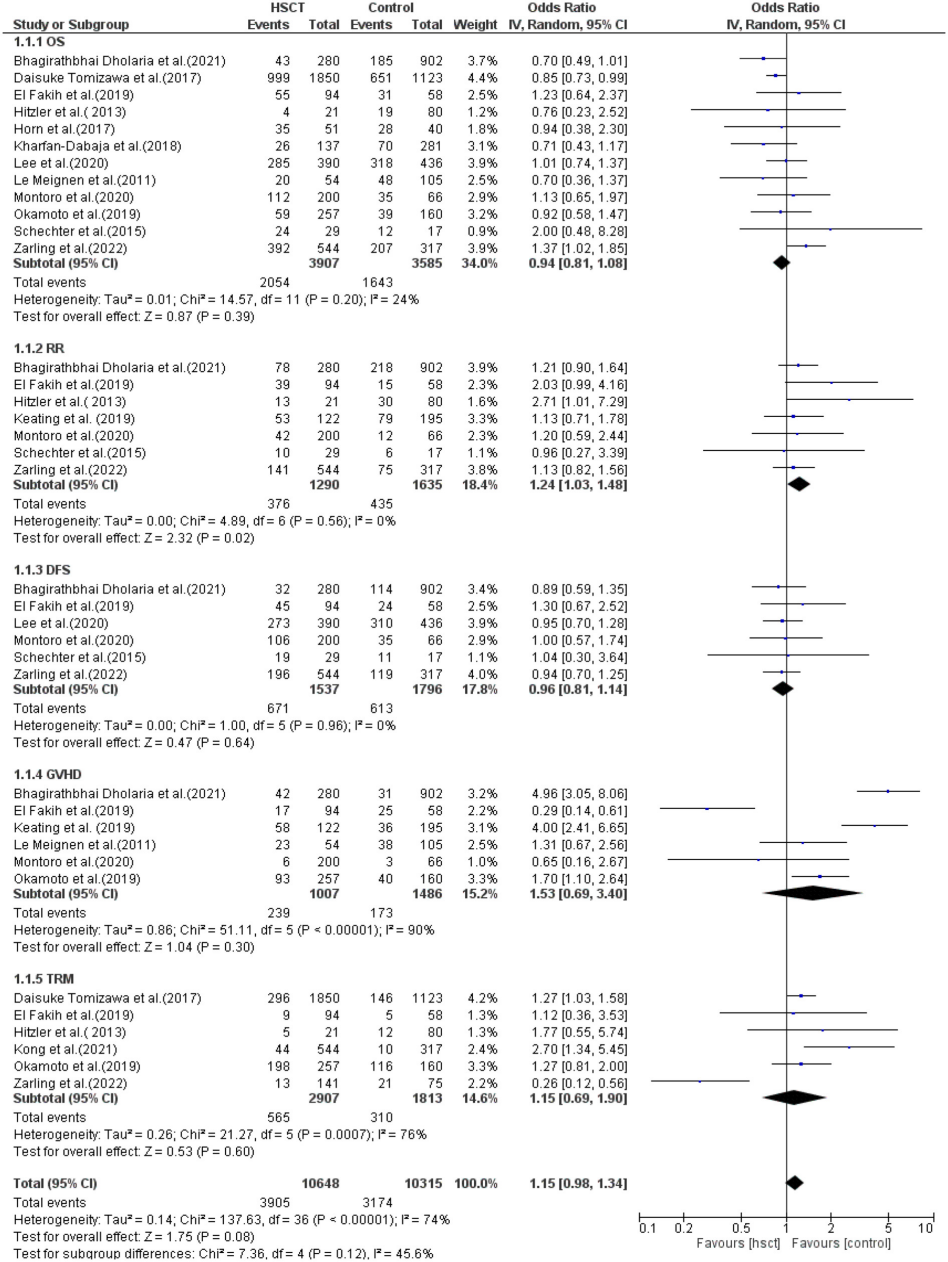
Forest plot for all the outcomes.

The reported outcomes were OS, RR, DFS, GVHD, and TRM. Data for OS was reported in 12 of the 14 studies included in the analysis. Heterogeneity among the studies for OS was not observed and did not reach statistical significance (Tau statistic = 0.01, *I*^2^ = 24%). Thus, a fixed-effects model was employed. Figure 3, section 1.1.1, displays a forest plot depicting the individual and pooled ORs, and through the aggregation of data from all the studies, we ascertained that there was a non-statistically significant difference between HSCT and the control intervention in terms of the OS of patients diagnosed with AML (pooled OR = 0.94; 95% CI, 0.81–1.08; *P* = 0.39, Figure 3).

RR was observed in seven studies, and heterogeneity was not reported among studies and did not reach statistical significance (Tau statistic = 0.00, *I*^2^ = 0%). Thus, a fixed-effects model was used. The forest plot in Figure 3, section 1.1.2, depicts the individual and pooled ORs, and through the aggregation of data from all the studies, we observed that there was a statistically significant difference between HSCT and the control intervention in terms of the RR of patients diagnosed with AML (pooled OR = 1.24; 95% CI, 1.03–1.48; *P* = 0.02, Figure 3).

DFS was reported in six of the 14 studies included in the analysis. Heterogeneity among the studies for DFS was not observed and did not reach statistical significance (Tau statistic = 0.00, *I*^2^ = 0%), so a fixed-effects model was used for analysis. Figure 3, section 1.1.3, displays a forest plot depicting the individual and pooled ORs. Through the aggregation of data from all the studies, we observed that there was a non-statistically significant difference between HSCT and the control intervention in terms of the disease-free survival of patients diagnosed with AML (pooled OR = 0.96; 95% CI, 0.81–1.14; *P* = 0.64, Figure 3).

GVHD was reported in six studies, and heterogeneity among the studies for GVHD was observed and reached statistical significance (Tau statistic = 0.86, *I*^2^ = 90%); thus, a random-effects model was applied for the analysis. Figure 3, section 1.1.4, displays a forest plot depicting the individual and pooled ORs. Through the aggregation of data from all the studies, we observed that there was a non-statistically significant difference between HSCT and the control intervention in terms of the graft-vs.-host disease of patients diagnosed with AML (pooled OR = 1.53; 95% CI, 0.69–3.40; *P* = 0.30, Figure 3).

**Figure 4:**
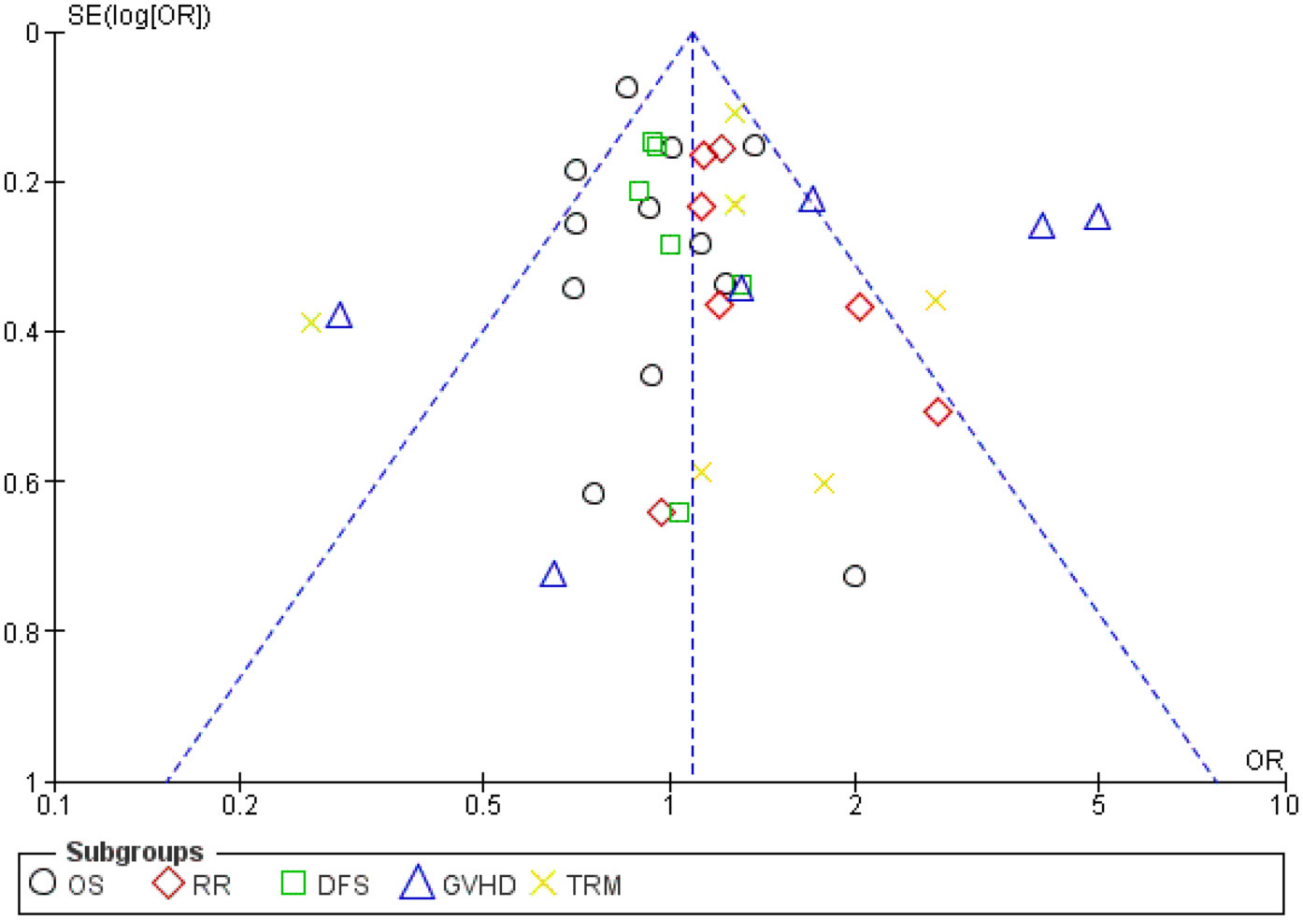
Funnel plot. OS, overall survival; TRM, treatment-related mortality; GVHD, graft-vs.-host-disease; DFS, disease-free survival; RR, relapse rate.

TRM was reported in six studies, and heterogeneity among studies was observed and reached statistical significance (Tau statistic = 0.26, *I*^2^ = 76%). Thus, a random-effects model was employed. Figure 3, section 1.1.5, displays a forest plot depicting the individual and pooled ORs, and through the aggregation of data from all the studies, we ascertained that there was a non-statistically significant difference between HSCT and the control intervention in terms of the TRM of patients diagnosed with AML (pooled OR = 1.15; 95% CI, 0.69–1.90; *P* = 0.60, Figure 3). Furthermore, the funnel plot (Figure 4) exhibited a symmetrical pattern, indicating no apparent publication bias.

### Subgroup analysis

The AML patients were grouped into adult and pediatric groups. Some studies reported a combination of pediatric and adult patients, so they were grouped together. The effectiveness of HSCT was compared to determine the group with the highest efficacy (Figure 4).

### Overall survival

**Figure 5:**
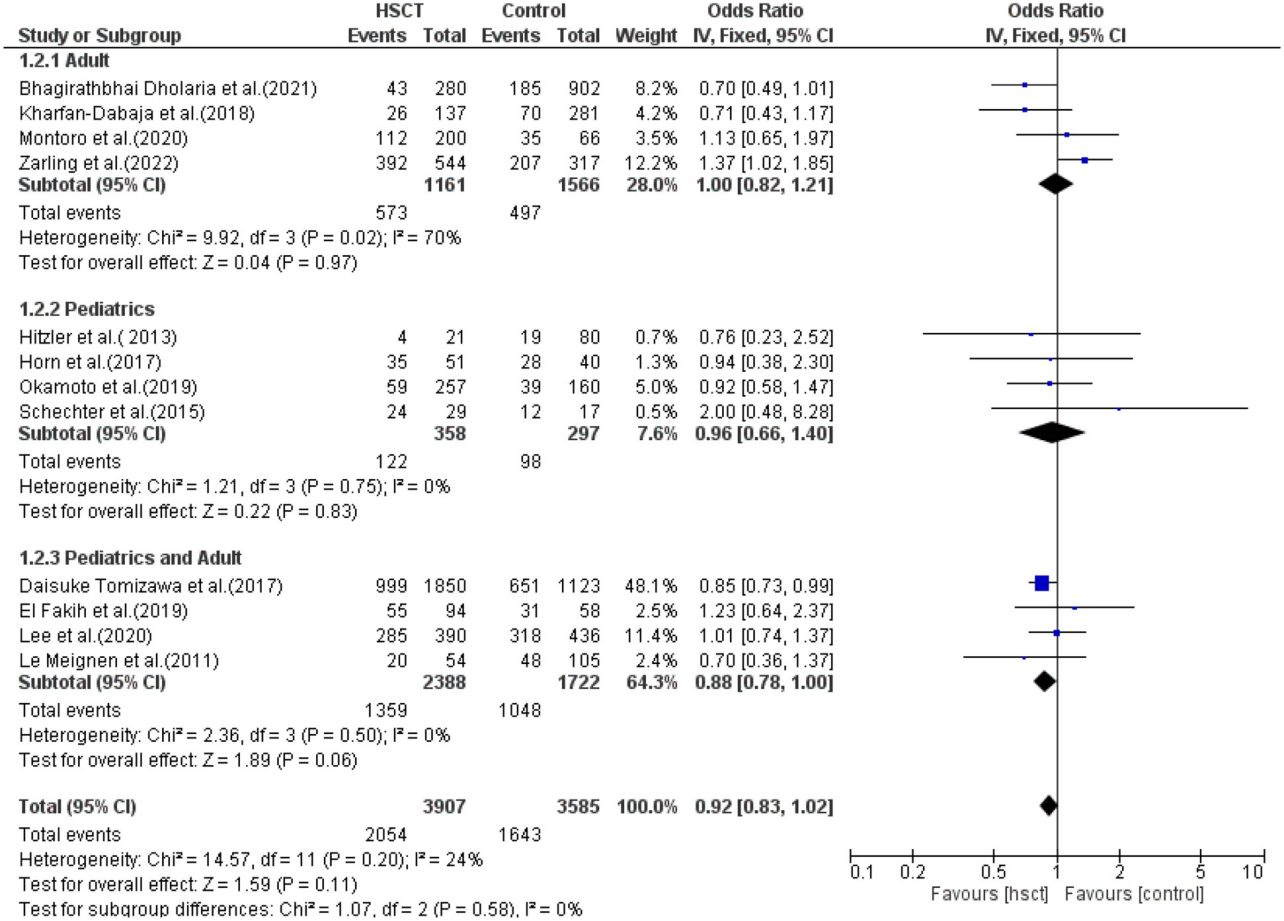
Subgroup forest plot for overall survival.

Four studies were included in the adult, pediatric, and young adult groups. The presence of heterogeneity was found and was significant only in the adult group (*I*^2^ = 24%, *P*-value = 0.02). The pooled ORs for the adult, pediatric, and combined pediatric and adult groups were 1.00 (95% CI: 0.82–1.21; *P* = 0.97), 0.96 (95% CI: 0.66–1.40; *P* = 0.83), and 0.88 (95% CI: 0.78–1.00; *P* = 0.06), respectively (Figure 5). The intervention was not found to be statistically different from the control intervention among the adult patients.

### Relapse rate

**Figure 6:**
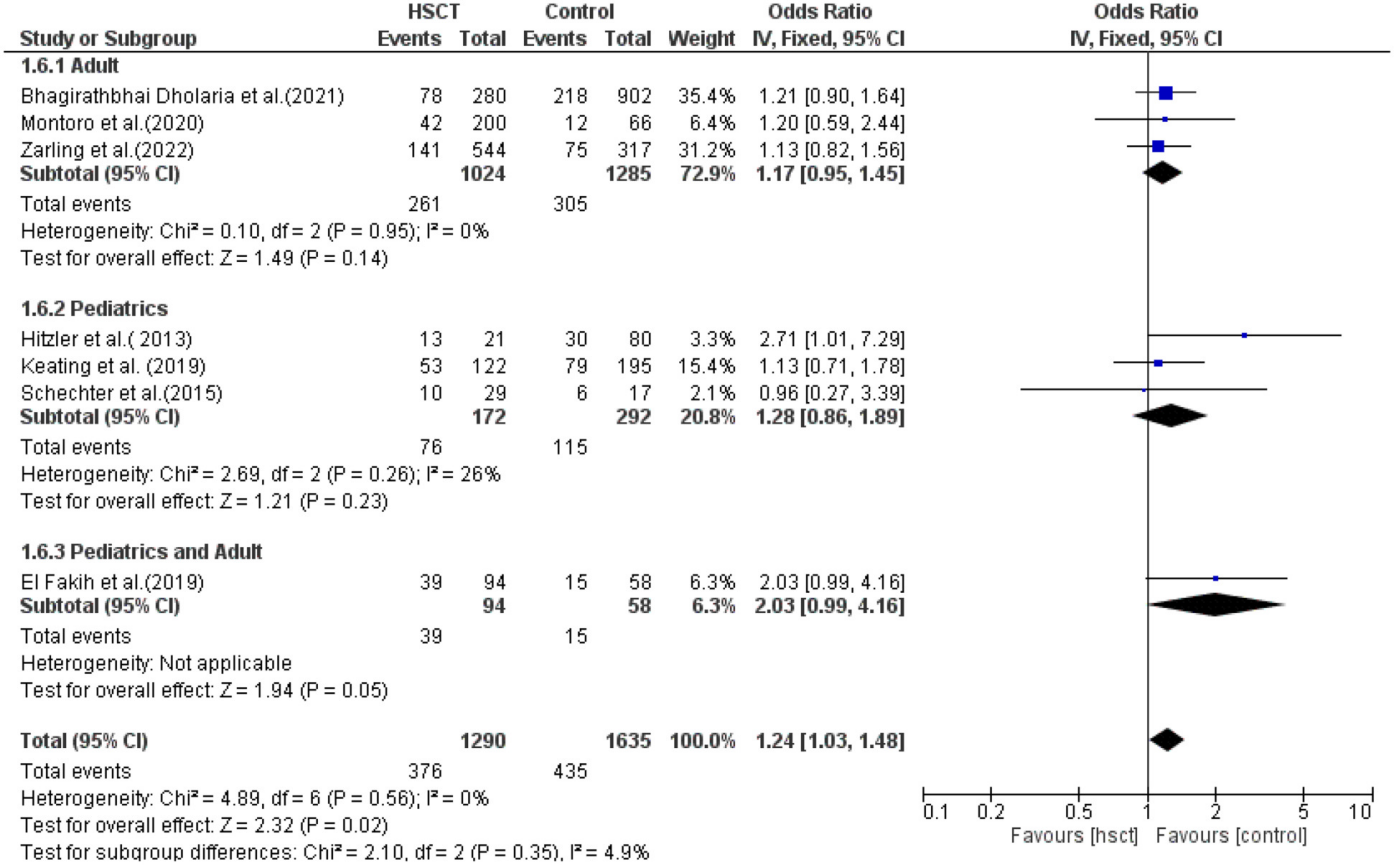
Subgroup forest plot for relapse rate.

Three studies were included in the adult group and pediatric group, while only one study reported young adults. There was no heterogeneity in the adult and pediatric groups (*I*^2^ = 0% and *I*^2^ = 6%, respectively). The pooled ORs for adult, pediatric, and combined pediatric and adult groups were 1.17 (95% CI: 0.95–1.45; *P* = 0.14), 1.28 (95% CI: 0.86–1.89; *P* = 0.23), and 2.03 (95% CI: 0.99–4.16; *P* = 0.05), respectively (Figure 6).

### Disease-free survival

**Figure 7:**
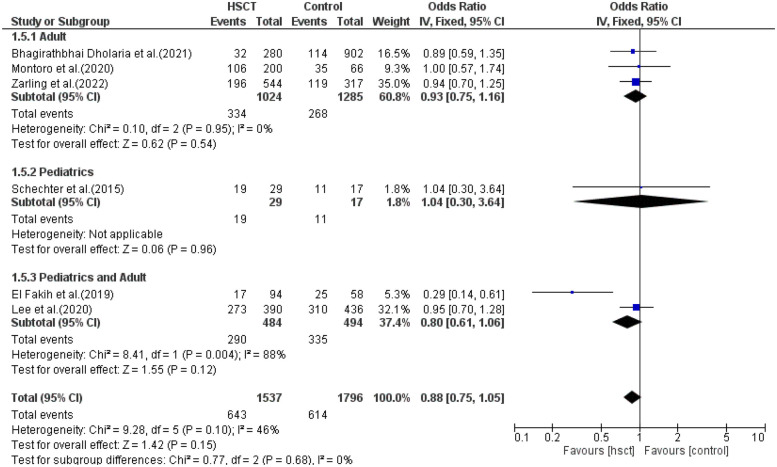
Subgroup forest plot for disease-free survival.

In the subgroup analysis of DFS, three studies were included in the adult group, only one in the pediatric group and two in the young adult group. The presence of heterogeneity was observed and was significant only in the young adult group (*I*^2^ = 88%, *P*-value = 0.004). The pooled ORs for the adult, pediatric, and combined pediatric and adult groups were 0.93 (95% CI: 0.75–1.16; *P* = 0.54), 1.04 (95% CI: 0.30–3.64; *P* = 0.96), and 0.80 (95% CI: 0.61–1.06; *P* = 0.12), respectively (Figure 7). The intervention was found not to have a statistical difference compared to the control intervention among the pediatric patients, and this result was not taken into consideration due to the presence of only one study in the pediatric group.

### Treatment-related mortality

**Figure 8:**
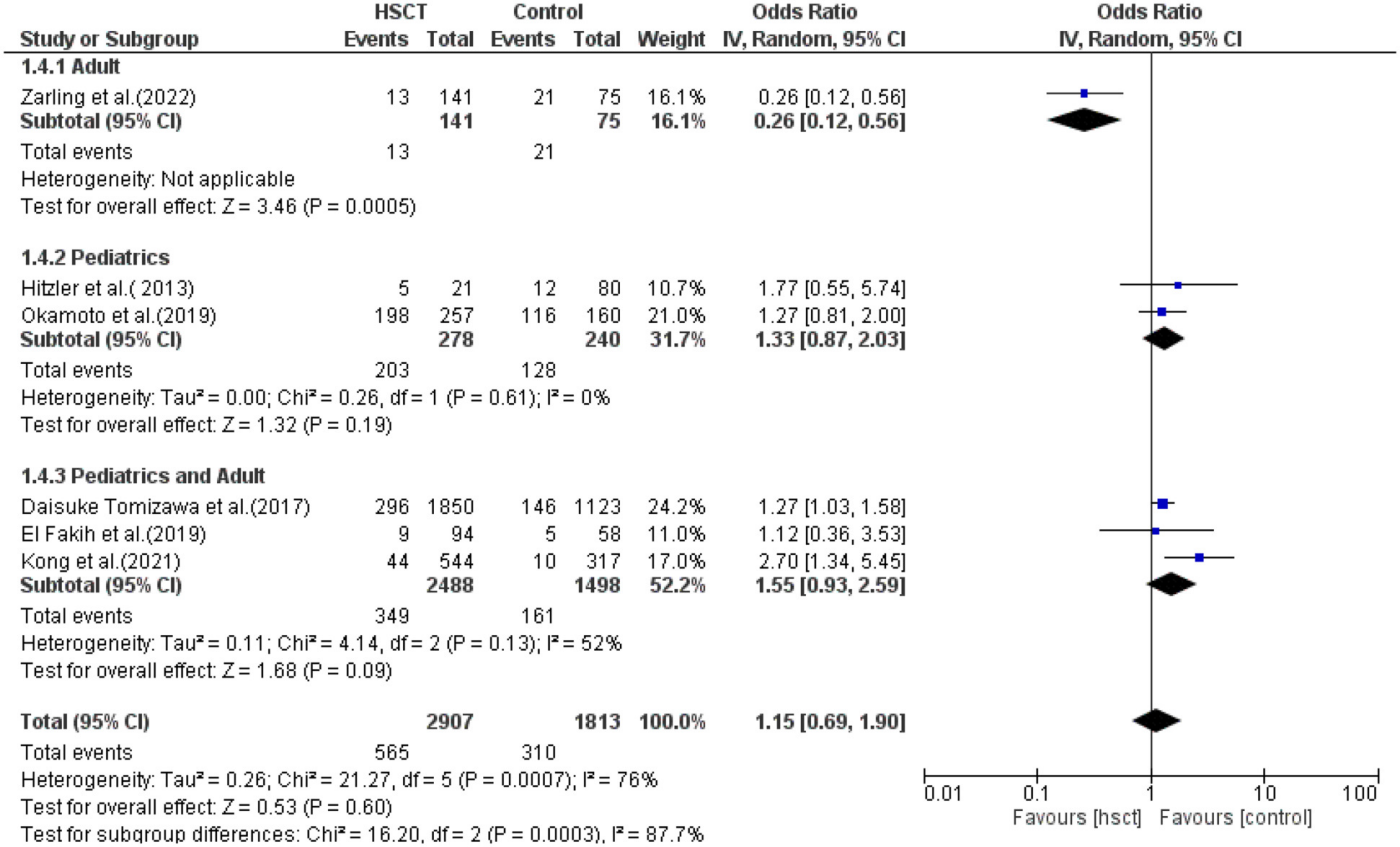
Subgroup forest plot for treatment-related mortality.

In subgroup analysis for TRM, only one study was included in the adult group, two in the pediatric group, and three in the pediatric + adult group. The presence of heterogeneity (moderate) was found and was significant only in the pediatrics + adult group (*I*^2^ = 52%, *P*-value = 0.13). The pooled ORs for adult, pediatric, and pediatric + adult groups were 0.26 (95% CI: 0.12–0.56; *P* = 0.0005), 1.33 (95% CI: 0.87–2.03; *P* = 0.19), and 1.55 (95% CI: 0.93–2.59; *P* = 0.09), respectively (Figure 8). The intervention did not have a statistical difference in TRM compared to the control intervention among the adult patients.

### Graft-vs.-host disease

**Figure 9:**
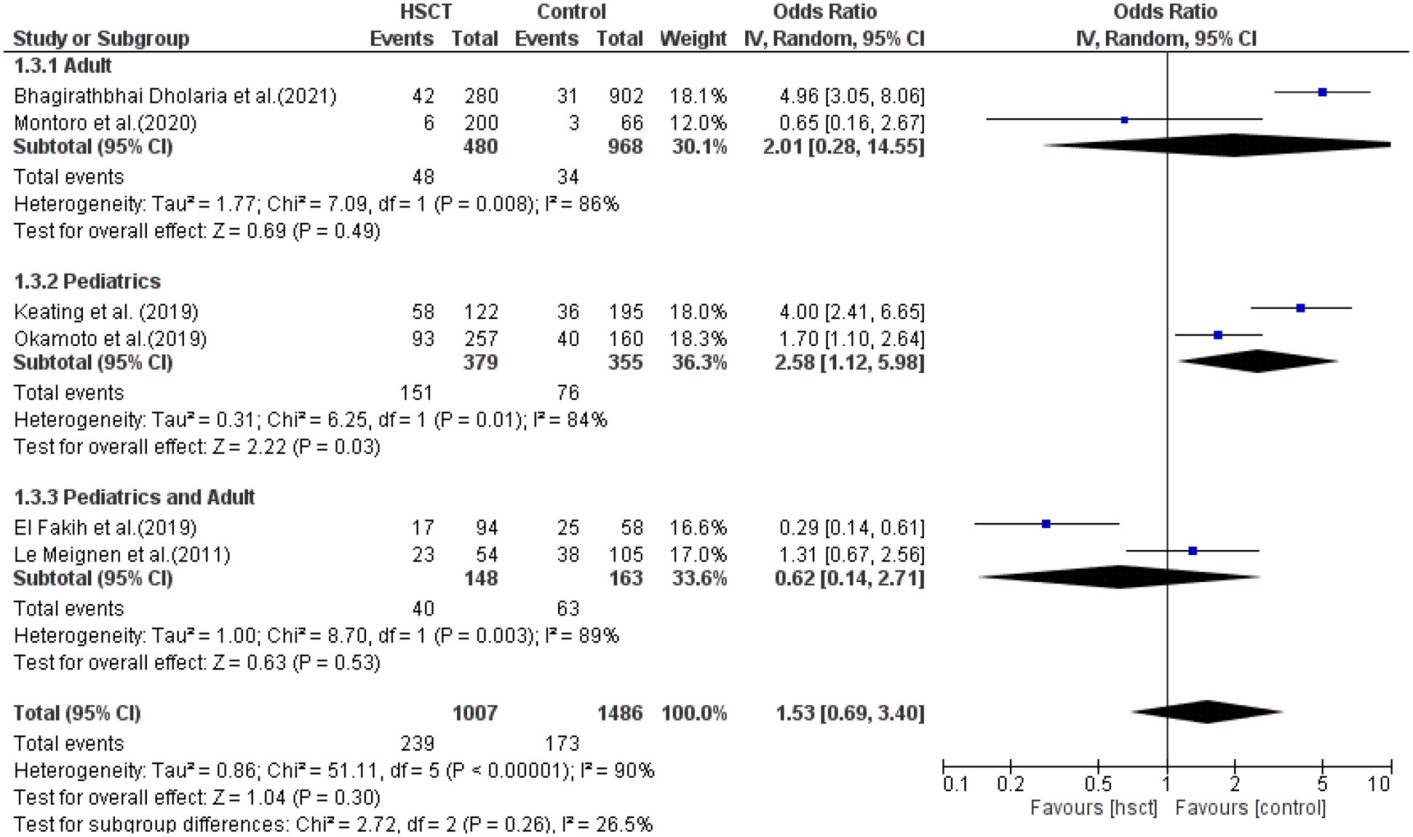
Subgroup forest plot for graft-vs.-host disease.

Only two studies were included in the adult, pediatric, and pediatric + adult groups. The presence of heterogeneity was found to be non-significant in the adult group (*I*^2^ = 86%, *P*-value = 0.008) and was significant in both the pediatric and pediatric + adult groups (*I*^2^ = 84%, *P*-value = 0.01; *I*^2^ = 90%, *P*-value <0.00001, respectively). The pooled ORs for the adult, pediatric, and pediatric + adult groups were 2.01 (95% CI: 0.28–14.55; *P* = 0.49), 2.58 (95% CI: 1.12–5.98; *P* = 0.03), and 0.62 (95% CI: 0.14–2.71; *P* = 0.53), respectively (Figure 9). The intervention was not statistically different in GVHD compared to the control intervention among the adult patients.

## Discussion

Insightful information on treatment outcomes was provided by comparing HSCT and other therapies in AML patients in two age group cohorts, i.e., adult and pediatric, in this study. In both age categories, HSCT proved to be effective by outperforming alternative treatments. This shows that HSCT, which offers the possibility of a cure and long-term disease control, is still a key component of AML treatment. The findings of this systematic review and meta-analysis support the efficacy of HSCT in pediatric AML. The analysis revealed that children undergoing HSCT experienced several beneficial outcomes. Most significantly, overall survival rates were encouraging, with a considerable proportion of patients surviving post-transplant without mortality or the onset of GVHD. This meta-analysis comparing HSCT outcomes in pediatric and adult patients with AML indicated several essential patterns. A statistically significant decrease was observed in TRM in the adult group (OR: 0.26, *P* = 0.0005), indicating the protective effect of HSCT in this subgroup analysis despite the higher baseline risk generally associated with comorbidities in older subjects. The pediatric group reported a statistically significant increase after HSCT (OR: 2.58, *P* = 0.03) in the incidence of GVHD, the possible reason for which may be increased immune responsiveness in pediatric patients. Other outcome variables, such as RR, OS, and DFS, showed positive trends in the pediatric and combined pediatric–adult groups when compared to the adult group alone. However, these differences did not reach statistical significance. Trends that do not attain statistical significance may offer valuable insights, particularly when they align with known biological or clinical factors. Several biological/clinical factors can explain the more positive outcomes reported in pediatric patients with AML undergoing HSCT. Children usually have fewer comorbid conditions and show greater resilience to myeloablative conditioning regimens, which may decrease transplant-induced toxicity (30).

Additionally, pediatric patients exhibit enhanced thymic function and more robust immune reconstitution following transplantation, which may contribute to a stronger GVL effect and a reduced risk of infection-related mortality (31, 32). Moreover, pediatric AML typically presents with a unique profile of molecular alterations and cytogenetic features, which can significantly impact the risk of relapse and responsiveness to treatment following HSCT (33). These factors collectively contribute to improved survival outcomes in pediatric cohorts. Adult patients with AML and comorbid conditions, such as diabetes, cardiovascular disease, and renal dysfunction, can have increased susceptibility to infection-related complications, organ toxicity, and delayed engraftment, thus further complicating transplant outcomes (34). Additionally, adults receive reduced-intensity conditioning regimens to decrease early mortality risk by minimizing toxicity, which may be related to higher relapse rates due to less potent cytoreduction (35). These factors collectively contribute to a complex balance between treatment efficacy, emphasizing the need for personalized therapy strategies based on comprehensive pre-transplant assessment tools such as the Hematopoietic Cell Transplantation–Comorbidity Index (HCT-CI).

Additionally, genetics/cytogenetic markers are essential in determining risk stratification and predicting outcomes following HSCT in AML. A study reported that favorable risk mutations, such as NPM1 without FLT3-ITD and biallelic CEBPA, are more prevalent and associated with better post-transplant survival (36). While adult patients with AML showed a higher burden of adverse-risk mutations, including FLT3-ITD, TP53 mutations, and complex karyotypes, these were linked to increased relapse and reduced overall survival post-HSCT (37). These genomic differences highlight the need to evaluate genetic profiling before transplant and tailor conditioning regimens and post-transplant monitoring accordingly. Recent studies have demonstrated that the stem cell factor (SCF), c-Kit receptor, and its ligand are essential in HSC maintenance, proliferation, and survival. Recent studies have reported that co-administration of SCF and NSC87877, a dual SHP-1/2 inhibitor, leads to synergistic activation of c-Kit signaling, promoting HSC proliferation and improving cellular responsiveness (38). These findings indicate that pharmacological interventions of c-Kit and its downstream pathways could serve as a promising therapy strategy to enhance HSC expansion and improve HSCT efficacy.

Our meta-analysis of HSCT results in adult patients with AML, however, revealed a more nuanced picture. Among adult patients with AML, we found differences in overall survival, recurrence rates, and treatment-related death. Notably, older persons, particularly those aged 60 and older, had increased treatment-related mortality, as demonstrated by Kong et al. (29), reflecting the difficulties associated with providing intense conditioning regimens and managing comorbidities in this population. The existence of harmful genetic abnormalities and complicated karyotypes also caused reduced survival rates and enhanced relapse rates in adult patients with AML. The ability of HSCT to treat adult patients with AML, especially those with favorable cytogenetic and molecular characteristics, has been proven. The choice to proceed with HSCT in older persons should be decided judiciously, considering the relative importance of the advantages and disadvantages.

It was found that pediatric patients in this meta-analysis had lower rates of relapse and treatment-related mortality and typically had better overall survival outcomes than adults. These results support previous studies and emphasize the unique biology and clinical traits of AML in different age groups. Pediatric patients frequently have more favorable genetic defects, and their capacity to withstand demanding treatment plans helps explain why their results are better.

This review is limited by the relatively small number of eligible studies and the heterogeneity in study design, patient populations, and outcome measures. To address this, we employed a narrative synthesis approach and conducted subgroup comparisons where feasible. Furthermore, a formal risk of bias assessment was performed using the Cochrane Risk of Bias tool for randomized controlled trials. The results of these assessments are presented in Figure 2 and were considered in the interpretation of the findings. Additionally, the eligible studies had non-uniform reporting of critical variables such as transplant type, stem cell source, conditioning regimens, and specific non-transplant therapies. These factors restricted our ability to perform detailed subgroup analyses and limited direct comparisons, particularly for outcomes such as GVHD, which are unique to transplant recipients. Another limitation is also important to discuss: while the adult group was analyzed as a whole, future studies should consider subdividing this population into young (20–45 years) and older adults (46–70 years), as this may help uncover age-specific variations in transplant outcomes and guide more personalized treatment planning.

In summary, HSCT is still an important therapeutic option for pediatric and adult patients with AML. Our study demonstrates age-related differences in outcomes, with pediatric patients showing more favorable outcomes and fewer transplant-related complications. Different biological and molecular factors likely influence these differences; a clear understanding of these factors can guide more personalized treatment regimens and inform future research focusing on extensive, multicenter cohort studies and randomized controlled trials to improve outcomes across all age groups.

Conclusively, it was discovered that pediatric patients showed a trend for better overall survival outcomes than adults, with a trend of decreased relapse rates and treatment-related mortality. Pediatric patients typically have more favorable genetic anomalies, and their capacity to withstand intense treatment regimens leads to better results. This study's limitations were the inability to find studies that directly compare the effect of HSCT in pediatric patients with that in adult patients and the smaller number of studies with pediatric patients. Overall, this study explored the benefits of HSCT in pediatric patients with AML compared to adult patients.

## Data Availability

The original contributions presented in the study are included in the article/Supplementary Material, further inquiries can be directed to the corresponding author.
